# Case Report: A Peculiar Case of Inflammatory Colitis After SARS-CoV-2 Infection

**DOI:** 10.3389/fimmu.2022.849140

**Published:** 2022-02-09

**Authors:** Mariangela Rutigliani, Matteo Bozzo, Andrea Barberis, Marco Greppi, Emanuela Anelli, Luca Castellaro, Alessandro Bonsignore, Antonio Azzinnaro, Silvia Pesce, Marco Filauro, Gian Andrea Rollandi, Patrizio Castagnola, Simona Candiani, Emanuela Marcenaro

**Affiliations:** ^1^ Department of Laboratory and Service - Histological and Anatomical Pathology Unit, Ente Ospedaliero (E.O.) Galliera Hospital, Genova, Italy; ^2^ Department of Experimental Medicine, University of Genoa, Genova, Italy; ^3^ Department of Abdominal Surgery - General and Hepatopancreatobiliary Surgery Unit, Ente Ospedaliero (E.O.) Galliera Hospital, Genova, Italy; ^4^ IRCCS Ospedale Policlinico San Martino, Genova, Italy; ^5^ Section of Legal and Forensic Medicine, Department of Health Sciences, University of Genoa, Genova, Italy; ^6^ Department of Earth, Environment and Life Sciences, University of Genoa, Genova, Italy

**Keywords:** COVID-19, SARS-CoV-2, necrotizing ulcerative colitis, cytotoxic immune cells, PD-1/PD-L1 axis

## Abstract

We report a case of inflammatory colitis after SARS-CoV-2 infection in a patient with no additional co-morbidity who died within three weeks of hospitalization. As it is becoming increasingly clear that SARS-CoV-2 infection can cause immunological alterations, we investigated the expression of the inhibitory checkpoint PD-1 and its ligand PD-L1 to explore the potential role of this axis in the break of self-tolerance. The presence of the SARS-CoV-2 virus in colon tissue was demonstrated by qRT-PCR and immunohistochemical localization of the nucleocapsid protein. Expression of lymphocyte markers, PD-1, and PD-L1 in colon tissue was investigated by IHC. SARS-CoV-2-immunoreactive cells were detected both in the ulcerated and non-ulcerated mucosal areas. Compared to healthy tissue, where PD-1 is weakly expressed and PD-L1 is absent, PD-1 and PD-L1 expression appears in the inflamed mucosal tissue, as expected, but was mainly confined to non-ulcerative areas. At the same time, these markers were virtually undetectable in areas of mucosal ulceration. Our data show an alteration of the PD-1/PD-L1 axis and suggest a link between SARS-CoV-2 infection and an aberrant autoinflammatory response due to concomitant breakdown of the PD-1/PD-L1 interaction leading to early death of the patient.

## Introduction

Novel coronavirus disease (COVID-19), caused by severe acute respiratory syndrome coronavirus-2 (SARS-CoV-2) infection, was first identified in late 2019 in Wuhan, China. Since then, it rapidly spread around the world and was declared a pandemic by the WHO on March 11, 2020. Interestingly, COVID-19 seems to be strangely and tragically selective, with only some of the infected people developing the illness. Although most critically ill COVID-19 patients are either elderly or have underlying medical problems such as cardiovascular disease, hypertension, diabetes mellitus, or cancer, some previously healthy and even relatively young individuals have died from COVID-19 (1). The pathophysiological consequences of SARS-CoV-2 infection on different organs are also variegate and still poorly understood. In particular, little data are available regarding the presence and histological localization of the virus in the gastrointestinal tract (2).

Herein, we report a case of inflammatory colitis after SARS-CoV-2 infection in a patient with no additional co-morbidity who died within three weeks of hospitalization. We documented the presence of the virus in colon tissue samples by both immunohistochemical (IHC) and molecular analysis and described the peculiar tissue damage and the characteristics of the immune infiltrate mainly related to the immune checkpoint/ligand expression pattern.

## Methods

Microscopic analysis was performed on formalin-fixed paraffin-embedded (FFPE) sections by hematoxylin-eosin (HE) staining. IHC staining was performed on 2-μm-thick paraffin sections with an automated IHC 623 staining system (Ventana BenchMark ULTRA, Ventana Medical Systems, Italy). anti-CD3 (clone 2GV6, prediluited, Ventana); anti-CD4 (clone SP35, prediluited, Ventana), anti-CD8 (clone SP57, prediluited, Ventana) anti-CD20 (clone L26, prediluited, Ventana), anti-CD138 (b-a38, prediluited Cell Marque), anti-CD68 (clone KP-1, prediluited, Ventana), anti-PD-1 (clone NAT105 prediluited, Cell Marque), anti-PD-L1 (clone SP263 prediluited, Ventana), NKp46 (clone 195314 prediluited, R&D Systems bio-techne catalogue number MAB1850). IHC localization of SARS-CoV2 was obtained using a polyclonal antibody anti-SARS nucleocapsid protein (NB100-56576, Novus Biologicals) and the DAKO Envision kit (Agilent). RNA was extracted from sections of formalin-fixed and paraffin-embedded (FFPE) colon tissue samples using the Qiagen RNeasy FFPE Kit following the manufacturer’s instructions. Real-time RT-PCR analysis for qualitative detection of SARS-CoV-2 nucleic acid was performed using the PerkinElmer New Coronavirus Nucleic Acid Detection Kit and the Roche LightCycler 480 Real-Time PCR System.

## Case Description

**Figure 1:**
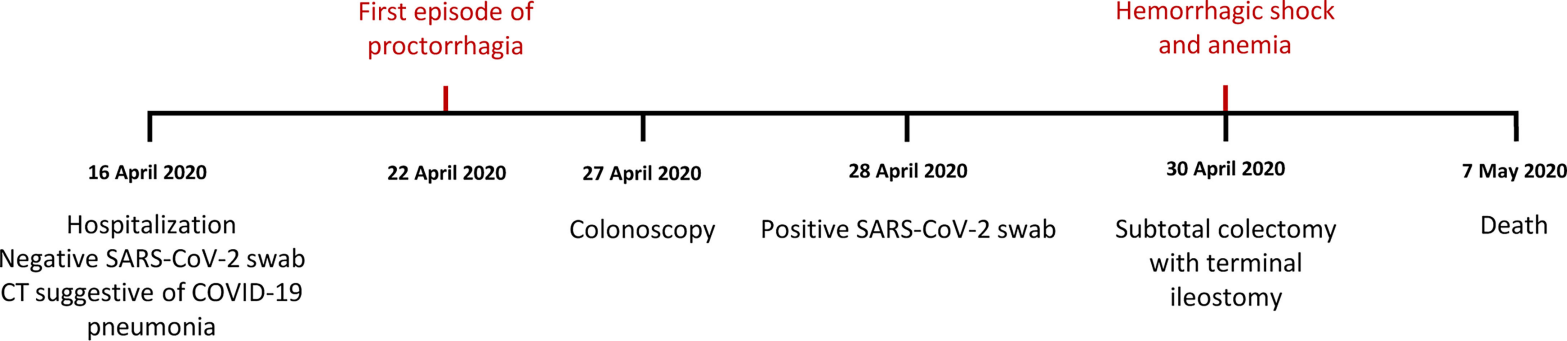
Timeline of the clinical case.

**Figure 2:**
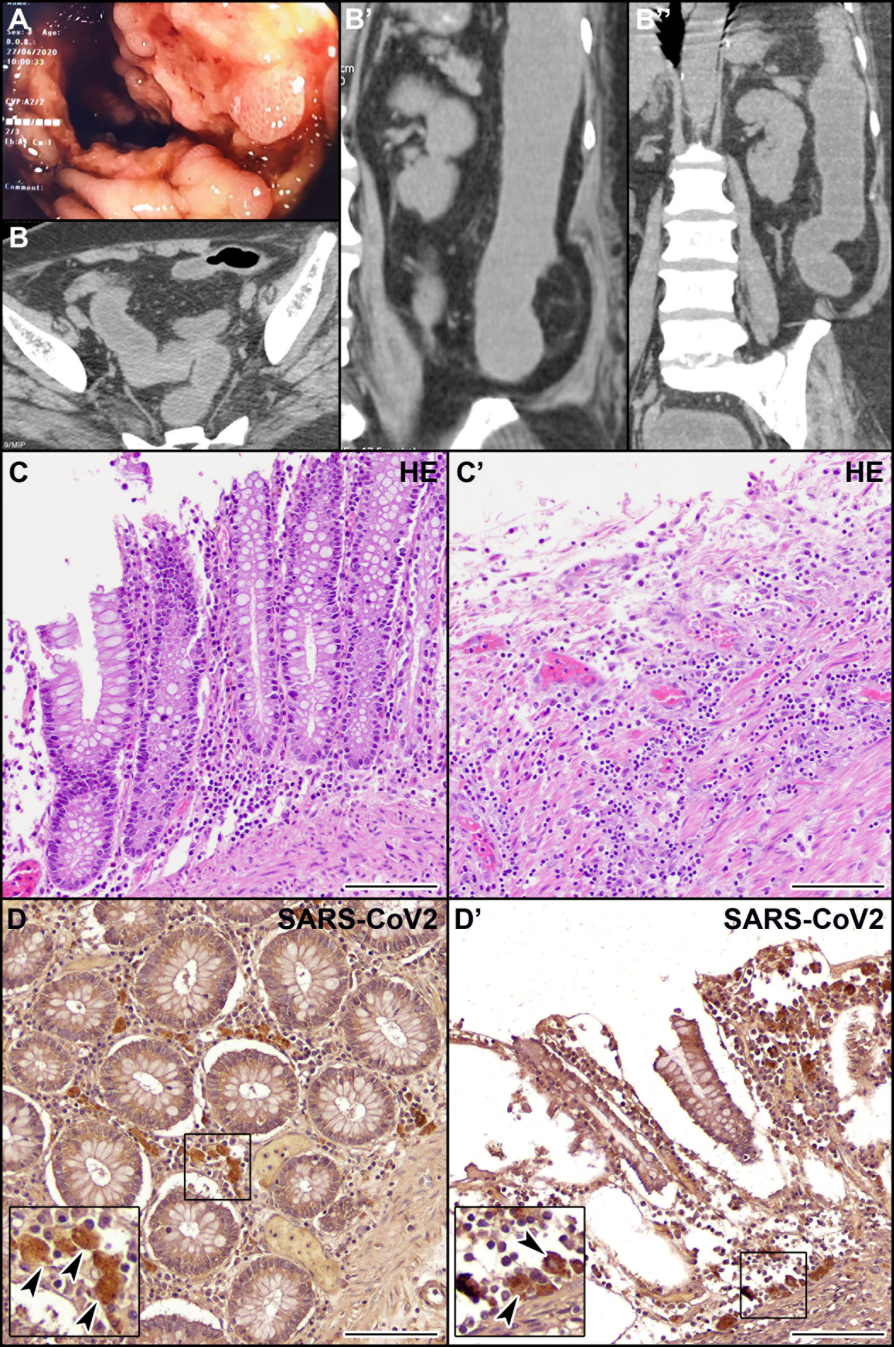
Endoscopic, CT, histological, and immunohistochemical (IHC) examination of the patient’s colon. **(A)** Endoscopic picture of the colon showing very severe inflammation with diffuse pseudopolyposis and ulcers. **(B, B’’)** CT scan of the abdomen in coronal **(B)** and axial **(B’, B’’)** planes reveals a stove-pipe colon with thinned wall and comb sign of the vasa recta indicative of acute inflammation. The rectum appears normal. **(C, C’)** Hematoxylin-eosin staining of non-ulcerated **(C)** and ulcerated **(C’)** areas of the colon mucosa. **(D, D’)** IHC localization of SARS-CoV-2 virus in macrophages (arrowheads) in the non-ulcerated **(D)** and ulcerated **(D’)** colon mucosa. Scale bars: 100 µm.

We report here the clinical case of a 71-year-old woman with no history of inflammatory bowel disease (IBD) who was hospitalized for dyspnea and sepsis. Blood tests showed a marked increase in C-reactive protein (38 mg/dl) and procalcitonin (26 ng/ml). Chest computed tomography (CT) scan showed findings suggestive of COVID-19 pneumonia. The SARS-CoV-2 oropharyngeal swab performed on admission was negative but became positive when it was repeated in the following days (**Figure 1**). The patient developed multiple episodes of proctorrhagia resulting in hemodynamic instability and need for multiple transfusions. Colonoscopy showed a very severe inflammation associated with pseudopolyps, ulceration (areas with acute ulcerative colitis), and diffuse bleeding (**Figure 2A**). The CT scan of the abdomen revealed a stove-pipe colon with thinned wall and comb sign of the vasa recta (**Figure 2B-B"**). After a hemorrhagic shock with anemia (Hb 5 g/L) the patient underwent surgery. A subtotal colectomy with terminal ileostomy was performed and, in the postoperative course, the clinical conditions rapidly deteriorated resulting in death five days post-surgery. The gross findings included a thickened sigmoid colon wall with serosal petechiae and necrotic-appearing splenic flexure and distal transverse colon. The histological examination of excised colon tissue specimens showed the presence of mucosal and submucosal ulcerations with lymphocyte infiltrate and numerous macrophages (**Figures 2C, C’**) and the presence of vasculitis pictures with occasional microthrombi and angiectasis with hemorrhagic spreading and edema in the mucosa and submucosa. The presence of SARS-CoV-2 in the excised colon tissue was confirmed by the detection of N protein mRNA by qRT-PCR (Ct=29). Consistent with this observation, IHC analyses revealed the presence of SARS-Cov-2-immunoreactive cells, likely macrophages, both in the ulcerated and non-ulcerated mucosal areas (**Figures 2D, D’**). This picture was consistent with the diagnosis of SARS-CoV-2-positive discontinuous necrotizing ischemic colitis with bowel perforation.

**Figure 3:**
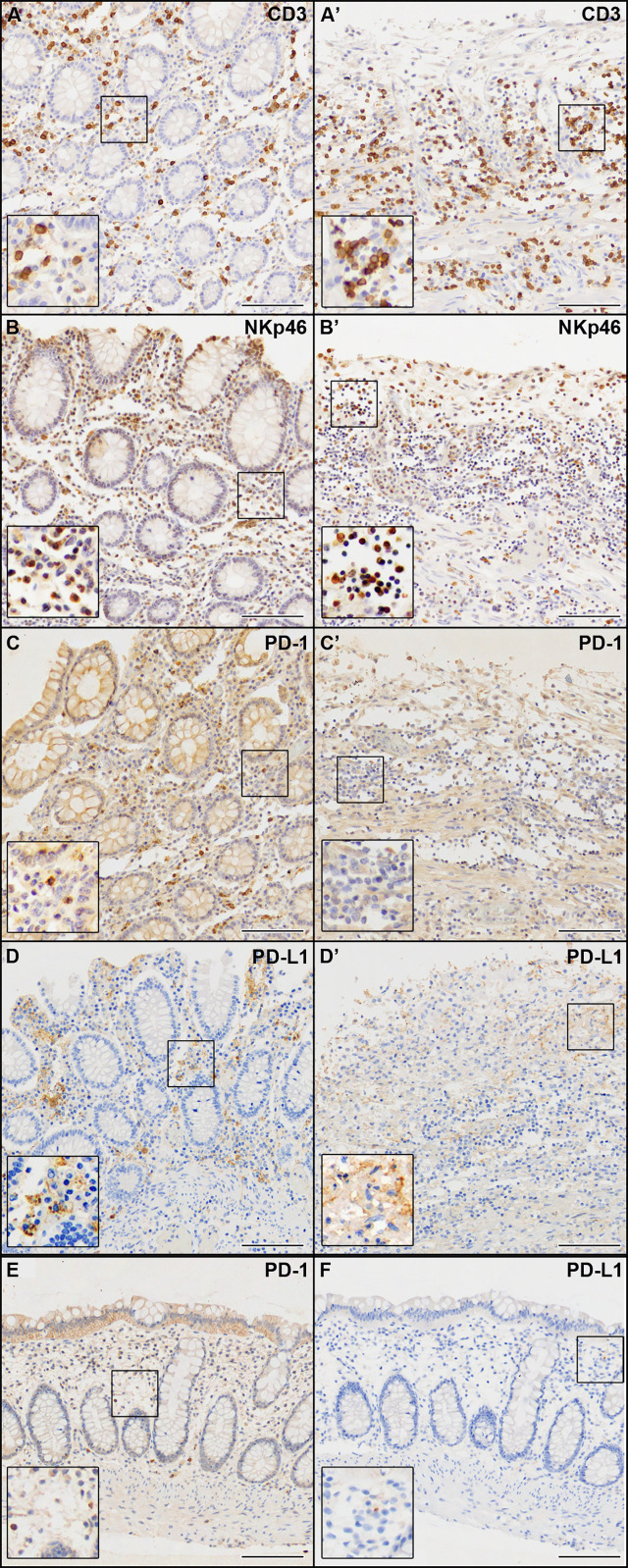
IHC analysis of the immune compartment markers indicated in each panel. **(A–D)** Non-ulcerated areas of the patient’s colon mucosa. **(A’–D’)** Ulcerated areas of the patient’s colon mucosa. **(E, F)** Colon mucosa from a healthy patient. Scale bars are 100 µm.

We observed the presence of abundant immune infiltrate both in the non-ulcerated mucosa and in areas of mucosal ulceration, mainly evident in the ulcerated colon tracts (**Figure 3**). IHC markers for the immune compartment revealed that the majority of infiltrating immune cells were CD3+ T cells (**Figures 3A, A’**), with a CD4:CD8 ratio of approximately 3:2 (data not shown). Abundant NKp46+ cells (Natural Killer cells of Group 3 Innate Lymphoid cells) were present (**Figures 3B, B’**). Few B lymphocytes CD20+ or plasma cells were found (<10% of all lymphocytes) (data not shown). Abundant (CD68+) macrophages were present (data not shown). As it is becoming increasingly clear that SARS-CoV-2 infection can cause immunological alterations (3), we also investigated the expression of the inhibitory checkpoint (IC) PD-1 and its ligand PD-L1 to explore the potential role of this axis in the break of self-tolerance and generation of necrotizing ischemic colitis with perforation of the bowel in some areas. We observed that the expression of PD-1 was mainly confined to immune cells infiltrating the non-ulcerative colon tissues (**Figures 3C, C’**). Interestingly, PD-L1 immunoreactivity was found in cells (primarily macrophages) infiltrating the non-ulcerative mucosal areas (**Figure 3D**), while this marker was virtually undetectable in areas of mucosal ulceration (**Figure 3D’**). Notably, under healthy conditions, we found a very weak immunoreactivity for PD-1 and no immunoreactivity for PD-L1 (**Figures 3E, F**). Although we cannot demonstrate that the absence of PD-L1 was due to SARS-CoV-2 infection, there seems to be a link between autoinflammatory diseases and SARS-CoV-2 infection.

## Discussion

COVID-19 pneumonia is a global disease caused by severe acute respiratory syndrome coronavirus 2 (SARS-CoV-2). Studies have increasingly reported the involvement of other organs in addition to the respiratory system, including the gastrointestinal tract. Indeed, the viral receptor angiotensin-converting enzyme-2 (ACE2) is highly expressed in terminal ileum and colon and the virus is often detected in stool samples (4, 5). It is not clear whether IBDs, characterized by a higher expression of ACE2 in the gastrointestinal tract, are a risk factor for SARS-CoV-2 infection or can negatively affect COVID-19 prognosis (6, 7). However, a few cases of ulcerative colitis, an IBD of unknown cause, resulting from SARS-CoV-2 infection have been reported (reviewed in 8).

Here we report a case of acute ulcerative colitis after SARS-CoV-2 infection in a 71-year-old female patient with no additional co-morbidity who died within three weeks of hospitalization. We observed an aberrant expression of the PD-1/PD-L1 markers in the colon mucosal tissue (high expression in the non-ulcerative mucosal areas and low/negative expression in the areas of mucosal ulceration). These data suggest that the acute autoinflammatory response observed during SARS-CoV-2 infection could be due to the disruption of the PD-1/PD-L1 interaction that resulted in the patient’s premature death. Indeed, it is of note that blockades of PD-1/PD-L1 interaction have been shown to lead to worsening autoimmune diseases and that PD-1 deficient mice develop autoimmunity (9). Anti-PD-L1 agents mainly act by restoring the effector function of cytotoxic (CD8+ T and NK) lymphocytes, which are also involved in defense against viral infections (10, 11). Considering the strict overlap between IC mechanisms and COVID-19 pathogenesis, a negative synergy in colon injury cannot be excluded.

The distinctive features of our case report, such as the hyper-acute clinical course with sudden deterioration, are uncommon for patients with COVID-19 and without co-morbidity. A possible explanation to the “explosive” clinical course observed could be that concomitant breakdown of the PD-1/PD-L1 interaction (an event reminiscent of IC treatment) and SARS-CoV-2 infection might have negatively synergized. This event has probably induced a hyper-activation of effector immune cells, promoting the so-called “cytokine-storm”, considered responsible for the severe acute respiratory distress syndrome in COVID-19 and in IC toxicity (12–14).

The histological case description, the viral detection in the colon, IC/IC ligands expression alterations and high levels of inflammatory markers support our hypothesis. To our knowledge, ours is the first case of COVID-19 ulcerative colitis in the literature describing a possible role for PD-1/PD-L1 axis. We believe that, in our patient, ulcerative colitis was triggered by COVID-19, as suggested by other case reports (15–17). From these data, it is clear that patients presenting with gastrointestinal complaints should also be evaluated for COVID-19, and further studies are needed to investigate whether COVID-19 predisposes to autoinflammatory diseases such as IBDs (especially ulcerative colitis) and also cancers such as colon cancer. The knowledge of pathophysiological mechanisms determined by SARS-CoV-2 infection can help to define better both the possible clinical course and the best therapeutic and pharmacological strategies to limit the evolution of the pathology. While waiting for further evidence on the risk of fatal colitis underlined by our case report, more intensive surveillance may be advisable for the role of IC regulators during the SARS-CoV-2 pandemic.

## Data Availability Statement

The raw data supporting the conclusions of this article will be made available by the authors, without undue reservation.

## Ethics Statement

Ethical review and approval was not required for the study on human participants in accordance with the local legislation and institutional requirements. Written informed consent for participation was not required for this study in accordance with the national legislation and the institutional requirements.

## Author Contributions

MR and EM (conceptualization, design, pathological examination, writing, supervision, editing manuscript). ABa (data collection, writing). GR, MF, and AA (data collection). ABo (editing manuscript). PC (molecular analyses, editing manuscript). SC, MB, SP, and MG (IHC analysis, editing manuscript). EA and LC (IHC analysis). All authors contributed to the article and approved the submitted version.

## Funding

The research leading to these results has received funding from AIRC under IG 2021 - ID. 26037 project – P.I. Marcenaro Emanuela. Additional grants: Italian Ministry of Health (PC), Compagnia di San Paolo n° 2019.866 (EM, SP, MB, SC, and MG); Roche per la Ricerca 2017 (EM and SP). MG was supported by a FIRC-AIRC fellowship for Italy.

## Conflict of Interest

The authors declare that the research was conducted in the absence of any commercial or financial relationships that could be construed as a potential conflict of interest.

## Publisher’s Note

All claims expressed in this article are solely those of the authors and do not necessarily represent those of their affiliated organizations, or those of the publisher, the editors and the reviewers. Any product that may be evaluated in this article, or claim that may be made by its manufacturer, is not guaranteed or endorsed by the publisher.
